# Meristem transitions and plant architecture—learning from domestication for crop breeding

**DOI:** 10.1093/plphys/kiab388

**Published:** 2021-08-26

**Authors:** Natalia Gaarslev, Gwen Swinnen, Sebastian Soyk

**Affiliations:** Center for Integrative Genomics, University of Lausanne, Lausanne, Switzerland

## Abstract

Genetic networks that regulate meristem transitions were recurrent targets of selection during crop domestication and allow fine-tuning of plant architecture for improved crop productivity.

Flowering plants display enormous architectural diversity that mainly results from differences in the position and organization of branches within vegetative and reproductive shoot systems. Where and when branches develop are tightly linked to the onset of flowering, which triggers the release of lateral buds from apical dominance and the outgrowth of additional branches. The transition to flowering depends on the activity of apical meristems, which are small groups of stem cells located at the growing tips of shoots. During vegetative growth, apical meristems produce vegetative organs including leaves and stem until endogenous and environmental signals prompt the transition to reproductive development, which often culminates in the production of an inflorescence, the flower-bearing shoot. It becomes clear that the rate at which meristems transition from the vegetative to the reproductive phase determines the number and pattern of branches in shoots and inflorescences (Figure 1). Not surprisingly, changes in shoot and inflorescence architecture have been selected during crop domestication to optimize the production of flowers, fruits, and seeds and remain a breeding target for crop improvement. In this update, we highlight examples of genes and genetic networks that regulate plant architecture in the model crop tomato (*Solanum lycopersicum*) and that were recurrent targets of selection during crop domestication and breeding. Fine-tuning the activity of conserved regulators of meristem transitions allows re-balancing vegetative to reproductive growth to customize plant architecture for improved crop productivity.

**Advances**

- Conserved regulators of meristem transitions were recurrent targets of crop domestication and breeding.
- Changes in the activity of conserved meristem regulators result in quantitative variation in shoot and inflorescence architecture.
- Tuning the expression of conserved meristem regulators by genome editing allows engineering plant architecture for crop improvement.
- Targeting conserved meristem regulators by genome editing facilitates the rapid improvement of underutilized crops and *de novo* domestication of wild species.
- Meristem maturation and plant architecture are omnigenic traits and dependent on genotypic context and environmental conditions.

**Figure 1:**
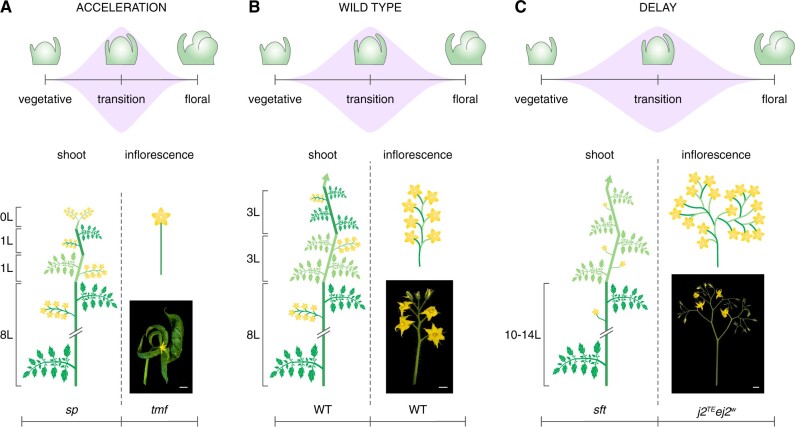
A model for how meristem maturation influences tomato plant architecture. Upper parts in (A) to (C) schematize meristem maturation scenarios to illustrate accelerated, timely, and delayed transition to flowering, respectively. Lower parts display the consequences on shoot and inflorescence architecture from the different maturation scenarios. A, Acceleration of floral transition leads to a reduced number of leaves on the primary shoot before the first inflorescence and a progressive decrease in leaf number on the sympodial shoot units (depicted in different shades of green) until sympodial shoot cycling stops. In the context of inflorescence architecture, a high rate of meristem maturation shortens the transient developmental window during which additional inflorescence meristems can be released (depicted by the purple graph) and results in a single-flowered inflorescence. B, Timely floral transition results in the production of seven to eight leaves on the primary shoot, continued sympodial shoot cycling with three leaves per sympodial unit, and multiflowered inflorescences with seven to eight flowers arranged on a single truss. C, Delays in floral transition increase the number of leaves on both the primary and sympodial shoots. With respect to inflorescences, a prolonged transient developmental window allows the production of additional lateral meristems giving rise to branched inflorescences. Scale bar, 1 cm; the number of leaves, L; diagrams show shoot architectures of *sp* (A), wild-type (WT) (B), and *sft* (C) plants; images depict detached inflorescences from *tmf* (A), WT (B), and *jointless2 enhancer-of-j2* (*j2^TE^ ej2^W^*) (C) plants.

## Meristem phase transitions shape plant architecture

**Figure 2:**
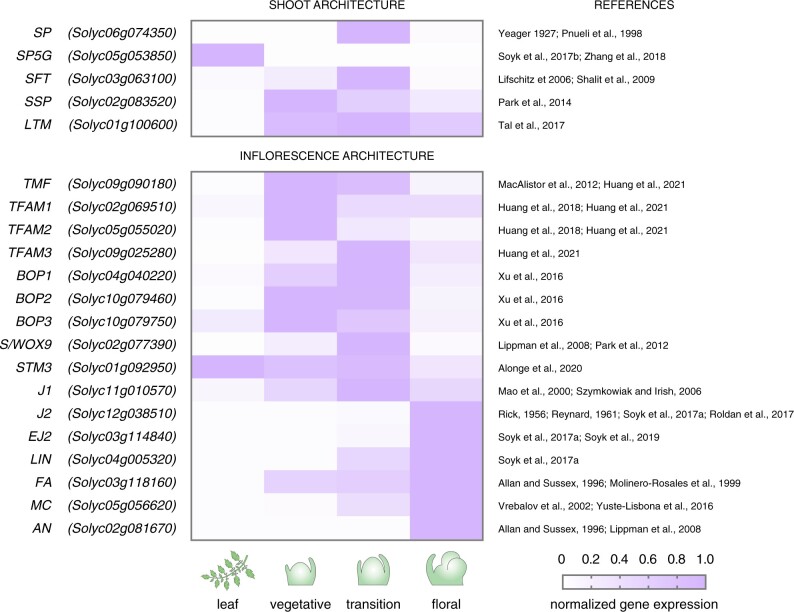
Dynamic expression of genes determining floral fate during meristem maturation. Shading represents expression relative to the highest expression value. Data were taken from the tomato meristem maturation atlas (Park et al., 2012) *SP, SELF PRUNING; SP5G, SELF PRUNING 5G; SFT, SINGLE FLOWER TRUSS; SSP, SUPPRESSOR OF SP; LTM, LATE TERMINATING MERISTEM*; *J*, *JOINTLESS*; *MC*, *MACROCALYX*; *S/WOX9*, *COMPOUND INFLORESCENCE/WUSCHEL-RELATED HOMEOBOX9*; *AN*, *ANANTHA*; *FA*, *FALSIFLORA*; *TMF, TERMINATING FLOWER; TFAM, TMF FAMILY MEMBER; BOP, BLADE ON PETIOLE*; *EJ2*, *ENHANCER OF JOINTLESS2; LIN, LONG INFLORESCENCE; STM3, SISTER OF TOMATO MADS-BOX3*.

The architectural diversity across species is especially vast for inflorescences, which come in uncountable shapes and sizes depending on the number and arrangement of flowers and branches (Castel et al., 2010). These species-specific differences were unified by mathematic modeling in a basic developmental concept, which proposes that variation in branching patterns depends on a hypothetical variable *vegetativeness* that changes gradually during plant development (Frijters, 1978; Prusinkiewicz et al., 2007). In this early model, high levels of *vegetativeness* refrain meristems from reproductive development, while low levels allow meristems to acquire floral fate. More recent models explain variation in branching patterns by changes in the rate of meristem maturation (Park et al., 2012, 2014a), meristem termination (Lifschitz et al., 2014; Meir et al., 2021), and meristem phase changes (Kyozuka et al., 2014), which propose the rate at which meristems transition between the vegetative and reproductive phase as a central variable. Although still under active debate, these models are corroborated by the genetic dissection of inflorescence mutants in multiple crop species, demonstrating that transitions between meristem phases are defined by stage-specific—yet fluent—patterns in gene expression and that subtle shifts in gene expression dynamics cause quantitative changes in inflorescence architecture (Figure 2; Park et al., 2012; Yoshida et al., 2013; Bommert and Whipple, 2018; Meir et al., 2021). Specifically, delays in the transition to floral fate allow apical meristems to continue with the production of additional lateral inflorescence meristems that result in branched, multiflowered inflorescences (Park et al., 2012; Yoshida et al., 2013; Soyk et al., 2017a). Conversely, accelerations in meristem maturation lead to faster floral termination and inflorescences with fewer flowers (MacAlister et al., 2012; Xu et al., 2016). This range of inflorescence complexity is represented within the *Solanaceae* family, which includes species with single-flowered (e.g. tobacco (*Nicotiana tabacum*) and pepper (*Capsicum annuum*)) and multiflowered inflorescences (e.g. tomato and related wild species). The evolution of *Solanaceae* inflorescence diversity involved changes in the rate of meristem maturation, which are driven by an increased transcriptional divergence of conserved regulatory genes during a critical developmental window that marks the transition to reproductive development (Lemmon et al., 2016). Recent advances in single-meristem genomics in tomato allowed a highly resolving dissection of the temporal events that occur during this developmental window (Meir et al., 2021). Transcriptome profiling of hundreds of individual meristems revealed that subtle morphological changes are accompanied by vast and rapid molecular events, and uncovered short-lived gene programs that are sequentially activated to guide the switch between meristematic states (Meir et al., 2021). Intriguingly, the dynamics in gene expression at meristem transitions display similarities to chemical reactions, in which an unstable intermediate state with high entropy separates states of low energy (Efroni, 2018; Omary et al., 2020). Similar principles are likely conserved across flowering plants and contribute to architectural diversity in other species given that the rate of meristem maturation affects inflorescence architecture beyond the *Solanaceae* (Kyozuka et al., 2014). Furthermore, the rate at which meristems transition from vegetative to reproductive growth affects the architecture of vegetative shoot systems in sympodial plants (Box 1) such as tomato and soybean, which continue vegetative growth after floral termination from lateral meristems (Figures 1 and 2; Tian et al., 2010; Jiang et al., 2013; Park et al., 2014b). As a result, it has been proposed that changes in the schedule of meristem maturation underlie quantitative variation in both shoot and inflorescence architecture across species (Park et al., 2014a; Meir et al., 2021).

Box 1 Monopodial versus sympodial growth habit

Plant architecture is defined by the number and disposition of vegetative and reproductive structures that are produced by the shoot apical meristem. During the vegetative phase, the apical meristem gives rise to stems and leaves. The floral transition prompts the meristem to enter the reproductive phase to produce flowers. Two main growth habits are described in flowering plants: monopodial and sympodial. In monopodial plants such as Arabidopsis, the apical meristem remains indeterminate after the transition to flowering and produces lateral floral meristems until being exhausted. In sympodial plants, such as tomato, the apical meristem is determinate and terminates in a flower while vegetative growth continues from a specialized axillary meristem (sympodial meristem) that is released in the axil of the last leaf. This process of floral termination and sympodial meristem release is reiterated indeterminately and results in the production of modular structures (sympodial units) that consist of a shoot with a terminal flower, resulting in a compound shoot that is characteristic for sympodial plants. In tomato, sympodial growth is recapitulated in inflorescences where each inflorescence meristem releases a sympodial inflorescence meristem at its flank before terminating in a flower, which results in the zig-zag arrangement of flowers on the tomato inflorescence.

## Switching from vegetative to reproductive phase—timing is key for achieving optimal shoot architecture

### How crop domestication and breeding altered shoot architecture

**Figure 3:**
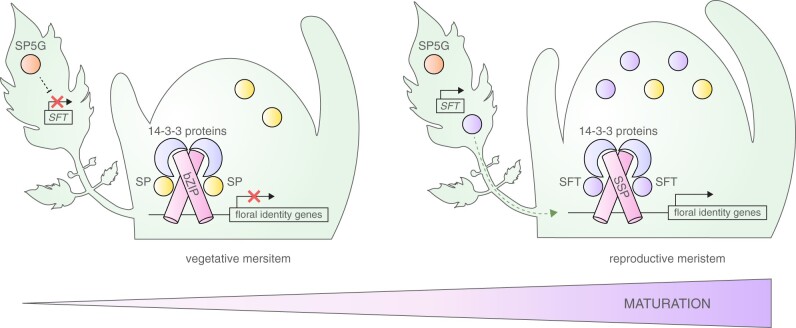
A model for how the florigen–antiflorigen ratio determines developmental stages of apical meristems. A, Florigen (SFT) and antiflorigen (SP) act antagonistically to regulate the transition of meristems from vegetative to reproductive growth by competing for bZIP transcription factors bound to the same *cis*-regulatory elements of floral identity genes. During long days, *SFT* expression in mature leaves is repressed by the activity of *SP5G*, allowing the formation of a floral repression complex in the apical meristem that consists of the antiflorigen SP and bZIP transcription factors, and is mediated by 14-3-3 scaffold proteins. During short days, *SP5G* expression is reduced which allows *SFT* expression and movement of the SFT protein to the apical meristem where it interacts with the bZIP transcription factor SSP through 14-3-3 proteins. The formation of this floral activation complex leads to the initiation of flowering.

Domestication and breeding of many crop species favored an increase in shoot determinacy (Box 2) to yield an architecture better suited for cultivation (Eshed and Lippman, 2019). Tomato is no exception and at the start of the 20th century, breeders discovered the spontaneous *self-pruning* (*sp*) mutation, which transformed indeterminate tomato vines into determinate bushes with concentrated fruit set that allows mechanical harvesting in large-scale field production (Yeager, 1927; Rick, 1978). Determinate growth of *sp* mutants is caused by sympodial meristems that progressively transition faster and terminate in flowers until sympodial shoot cycling completely stalls (Pnueli et al., 1998). Sympodial meristems are normally refrained from acquiring floral fate by the antiflorigenic activity of *SP*, which is a homolog of Arabidopsis *TERMINATING FLOWER1* (*TFL1*) and belongs to the *CETS* (*CENTRORADIALIS* [*CEN*], *TFL1*, *SP*) gene family (Figure 3; Pnueli et al., 1998). *SP* encodes an antiflorigen that acts as a repressor of flowering and antagonizes the activity of *SINGLE FLOWER TRUSS* (*SFT*), another *CETS* gene and homolog of Arabidopsis *FLOWERING LOCUS T* (*FT*). *SFT* encodes the universal flowering hormone florigen and triggers the transition of meristems to reproductive growth (Lifschitz et al., 2006; Shalit et al., 2009). The floral transition goes along with a gradual expansion and doming of the apical meristem, which is coordinated by the kelch repeat protein LATE TERMINATING MERISTEM (LTM) (Tal et al., 2017). In the absence of LTM, meristems dome early and express *SP* precociously, indicating that *LTM* activity suppresses *SP* expression in vegetative meristems. Although *ltm* mutants undergo the morphological changes of the floral transition earlier, they flower late. Therefore, *LTM* is suggested to protect meristems from floral termination under strong flowering signals by synchronizing *SP* expression. Since *ltm* and* sft* mutations are additive, *LTM* likely coordinates the floral transition independent of florigen (Tal et al., 2017).

Box 2 The concept of meristem determinacy

The fate and timing of organ development throughout the lifecycle of a plant depend on the activity of meristems. The level of meristem determinacy defines the number of organs that a meristem produces, while meristem identity determines the type of organs that arise. During vegetative meristem stages, meristems give rise to leaves and stem until they transition to reproductive stages to give rise to inflorescences and flowers. As a result, the level of meristem determinacy defines plant architecture by dictating the spatial and temporal patterns of organ development.

When endogenous and environmental signals favor flowering and reproduction, *SFT* expression is induced in the phloem companion cells of mature leaves (Lifschitz et al., 2006; Shalit et al., 2009). The SFT protein moves to apical meristems where it functions as a transcriptional regulator by interacting with the basic region leucine zipper (bZIP) transcription factor SUPPRESSOR OF SP (SSP), a homolog of Arabidopsis FLOWERING LOCUS D (FD) (Wigge et al., 2005; Park et al., 2014b; Pnueli et al., 2001). Mobile florigens also affect flowering-independent developmental processes and have been shown to regulate vascular development in tomatoes to coordinate vegetative and reproductive growth (Lifschitz et al., 2014; Shalit-Kaneh et al., 2019). At the shoot apex, florigen forms a floral activation complex with bZIP transcription factors that are mediated by 14-3-3 scaffold proteins, and evidence from yeast suggests that the protein complex is conserved in rice and tomato (Pnueli et al., 2001; Taoka et al., 2011; Park et al., 2014b). Floral activation and repressing complexes, which contain SFT or SP, are believed to regulate the expression of floral identity genes to ensure a timely transition to flowering. However, meristems eventually transition even in the complete absence of *SFT* activity although no other functional *SFT* orthologs have been identified in tomato (Lifschitz et al., 2014). The late transition of *sft* mutants requires the activity of the floral specification factor *FALSIFLORA* (*FA*), the ortholog of Arabidopsis *LEAFY* (Molinero-Rosales et al., 1999). Both *sft* and* fa* single mutants flower extremely late while *sft fa* double mutants never flower (Molinero-Rosales et al., 2004). Therefore, it has been proposed that *SFT* and* FA* function in parallel pathways but it is still insufficiently understood how *FA* and the florigen pathway are integrated and how other *CETS* genes might contribute (Molinero-Rosales et al., 2004; Lifschitz et al., 2014).

**Figure 4:**
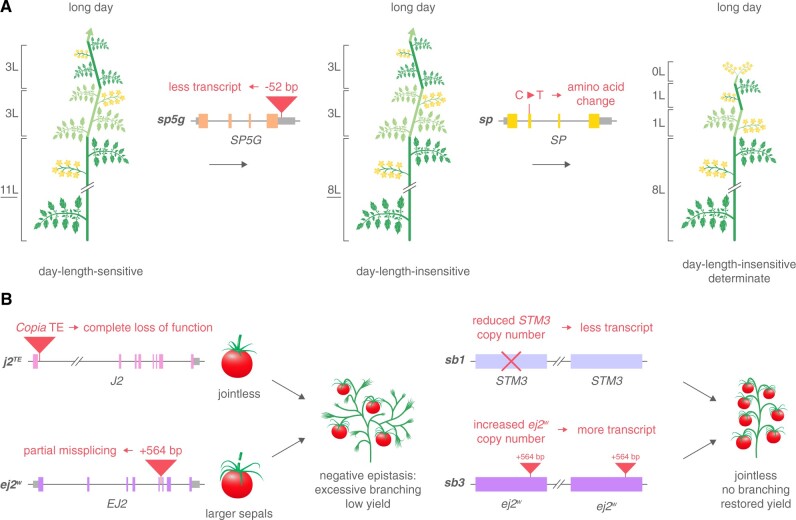
Genes and alleles that shaped shoot and inflorescence architecture during tomato domestication and breeding. A, Mutations in genes affecting day-length sensitivity and shoot determinacy yielded an architecture better suited for cultivation. A *cis*-regulatory mutation, which causes a reduction in *SP5G* transcript in long days, leads to the loss of day-length-sensitive flowering. A missense mutation in *SP* results in determinate growth. Together, both mutations lead to day-length-insensitive and determinate tomato plants. The number of leaves, L. B, Negative epistasis on fruit yield was overcome by selecting genetic suppressors. An intronic insertion of a *Copia* transposable element (TE) in *JOINTLESS2* (*J2*) causes a desirable jointless pedicel that improves harvesting. Introducing the *j2^TE^* allele in backgrounds carrying the weak *enhancer of jointless2* (*ej2^w^*) mutation, which leads to a reduction in functional transcript level because of partial missplicing, results in excessive inflorescence branching and low fertility. The *suppressor of branching1* (*sb1*) and *sb3* loci were selected to suppress negative epistasis between *j2^TE^ and ej2^w^*. A reduction in *STM3* copy number explains *sb1* and leads to a decrease in *STM3* transcript level, while an increase in *ej2^w^* copy number explains *sb3* and results in an increase of functional *EJ2* transcript. Gene models: exons, untranslated regions, and insertions are indicated by colored boxes, gray boxes, and red triangles, respectively.

The determinate growth habit from the *sp* mutation depends on the genetic background and is less severe in genotypes that harbor a functional allele of* SELF PRUNING 5G* (*SP5G*), a flowering repressor that belongs to the *CETS* gene family (Eshed and Zamir, 1995; Jones et al., 2007; Soyk et al., 2017b). Functional alleles of *SP5G* are found in wild tomato relatives that are native to regions near the equator in South America. In short days, the direct ancestor of tomato (*S. pimpinellifolium*) and other closely related wild species rapidly transition to flowering (Soyk et al., 2017b; Song et al., 2020). However, in long days such genotypes produce highly vegetative shoots because flowering is delayed by high *SP5G* activity (Figure 4). In long photoperiods, *SP5G* is upregulated and functions as a repressor of flowering by reducing the expression of *SFT* in mature leaves (Soyk et al., 2017b). This response to day-length is strongly mitigated in domesticated tomato by a *cis*-regulatory mutation downstream of *SP5G*, resulting in lower *SP5G* expression and consequently near-day-neutral flowering (Zhang et al., 2018). Furthermore, the activity of the closely related *SP5G* homolog *FT-LIKE1* (*FTL1*) is associated with higher *SFT* expression and accelerated flowering in short days (Cao et al., 2016; Song et al., 2020). The near-complete loss of day-length sensitivity resulting from mutations in *SP5G* and* FTL1* facilitated tomato cultivation in geographic regions away from the equator. In addition, loss of *SP5G* activity has been a prerequisite for the utilization of determinate *sp* varieties in field production (Figure 4; Jones et al., 2007).

Relative changes in the ratio of florigen-to-antiflorigen rather than absolute levels determine the rate by which meristems transition and terminate (Lifschitz et al., 2014). In *sft* mutants, florigenic signals are reduced and the antiflorigenic activity of *SP* dominates, thereby delaying meristems from acquiring floral fate. This results in late flowering and highly vegetative plants with poor fruit yields (Krieger et al., 2010). Conversely, lower levels of antiflorigen in *sp* mutants allow strong florigenic signals to induce flowering precociously. Although the florigen–antiflorigen model has been described across flowering plants, it is insufficiently understood at the molecular level. Recent findings in Arabidopsis suggest that antagonism between florigen (FT) and antiflorigen (TFL1) takes place through competition for bZIP transcription factors that are bound to *cis*-regulatory elements of floral identity genes (Jaeger et al., 2013; Zhu et al., 2020). However, it remains to be determined how florigen–antiflorigen ratios are established and relayed to changes in gene expression, and if transcription factors other than FD/SSP and related bZIP factors are involved in floral activation and repression complexes.

### Optimizing crop architecture by tuning the florigen-antiflorigen ratio

The florigen–antiflorigen system regulates shoot architecture in a quantitative and gene dosage-dependent manner. This is illustrated by natural *sp* mutants, which carry a missense mutation of moderate effect and are less determinate and higher yielding than clustered regularly interspaced short palindromic repeat (CRISPR)-engineered *sp* null mutants (Pnueli et al., 1998; Lemmon et al., 2018). The dosage relationship has been exploited by genome editing of the regulatory regions to fine-tune *SP* expression. Engineering *cis*-regulatory alleles of *SP* allowed the generation of novel *sp* genotypes that show a quantitative range of shoot determinacy (Rodríguez-Leal et al., 2017). Further reduction in antiflorigenic signals by simultaneously mutating *SP* and* SP5G* by genome editing accelerates flowering on all shoots and results in compact varieties with early fruit sets (Soyk et al., 2017b). The earliness for yield from *sp sp5g* can be stacked with compact growth from mutations in the *ERECTA* homolog of tomato (*SlER*) to obtain highly compact and early yielding varieties that are optimized for indoor cultivation (Kwon et al., 2020). Tipping the balance in favor of antiflorigen with heterozygous loss-of-function mutations in *SFT* or the interacting bZIP transcription factor gene *SSP* reduces shoot determinacy in *sp* backgrounds, leading to the production of additional shoot units and inflorescences (Krieger et al., 2010; Jiang et al., 2013; Park et al., 2014b). This demonstrates that adjusting the florigen–antiflorigen ratio allows optimization of tomato plant architecture for yield improvements. Tuning shoot architecture by modulating the florigen–antiflorigen ratio has been also realized in other *Solanaceae* crop species, although species-specific differences have been observed. For example, targeting the closest homolog of *SP* in groundcherry (*Physalis grisea*) by genome editing transforms sympodial meristems into inflorescence meristems that terminate in single flowers (Lemmon et al., 2018), while loss of the *SP* homolog in pepper causes the production of flower clusters due to rapid termination of all sympodial shoot units (Kim et al., 2006; Elitzur et al., 2009). Such differences likely result from species-specific florigen–antiflorigen ratios and redundancy with additional *CETS* gene family members.

Improvements of plant architecture and crop productivity from re-calibrating the balance between florigenic and antiflorigenic signals are not limited to *Solanaceae* species. The florigen–antiflorigen system is conserved in flowering plants and has been a recurrent target during the domestication and breeding of many crop species. Examples include natural mutations in *SP* homologs that have been selected in soybean, common bean, mung bean, and sunflower to convert indeterminate wild progenitors into determinate domesticates (Tian et al., 2010; Blackman et al., 2010; Repinski et al., 2012; Li et al., 2018a). Furthermore, induced mutations in *SP* homologs in crops as diverse as cotton, cucumber, strawberry, and kiwi affect the balance between vegetative and reproductive growth and promise improvements in crop performance (Gaston et al., 2020; Wen et al., 2019; Varkonyi‐Gasic et al., 2019; McGarry and Ayre, 2021). Genome editing will enable a precise modification of florigen–antiflorigen ratios and is poised to fast-forward breeding programs for improved shoot architecture in under-utilized crops and even facilitate the *de novo* domestication of wild species (Lemmon et al., 2018; Zsögön et al., 2018; Li et al., 2018b; Eshed and Lippman, 2019).

## Optimizing inflorescence architecture by tuning the rate of meristem maturation

### Changes in inflorescence architecture during tomato domestication and breeding

While the architecture of tomato shoots dramatically changed from the *sp* mutation, inflorescences remained largely unaffected during tomato domestication and breeding (Rick, 1978). Most wild tomato ancestors and modern tomato cultivars develop inflorescences that consist of a single pseudo-branch on which flowers are arranged in a zigzag pattern (Figure 1; Peralta and Spooner, 2005). Domestication and breeding brought only subtle variation to this scheme in some cultivars that develop weakly branched inflorescences (Mata-Nicolás et al., 2020). However, wild and domesticated tomato display striking differences in the number of flowers per inflorescences. The wild ancestor species *S. pimpinellifolium* develops inflorescences with more than twice the number of flowers compared with domesticated types. The genetic architecture of flower number variation was dissected by classical linkage mapping, which revealed a polygenic nature of the trait (Grandillo and Tanksley, 1996; Doganlar et al., 2002; Van Der Knaap and Tanksley, 2003). Although the causative gene variants still remain to be identified it has been suggested that the number of flowers per inflorescence is affected by the antiflorigen *SP* (Grandillo and Tanksley, 1996). Effects of *SP* activity on inflorescence architecture become evident when the floral identity genes *JOINTLESS1* (*J1*) and *MACROCALYX* (*MC*) are mutated (Szymkowiak and Irish, 2006; Shalit et al., 2009). Single *j1* and* mc* mutants develop inflorescences with several flowers but then revert to vegetative growth, which results in leafy inflorescences (Lifschitz et al., 2014). However, the *sp* mutation suppresses inflorescence reversion in *j1 sp* and* mc sp* double mutants, indicating that loss of *SP* activity increase inflorescence meristem determinacy. Similar effects of the florigen–antiflorigen system on inflorescence development have been also reported in mutants with reduced florigen levels. Complete loss of *SFT* activity in *sft* mutants results in inflorescences that revert to vegetative growth after producing a single flower, while *sft* heterozygotes produce slightly more flowers per inflorescence (Shalit et al., 2009; Krieger et al., 2010). Overall, these studies suggest that the florigen–antiflorigen system is involved in regulating inflorescence meristem determinacy. However, it remains unclear how the effect of florigen–antiflorigen ratios differs between vegetative and inflorescences meristems (see “Outstanding Questions”). Characterization of additional *CETS* members and interacting partners might allow the uncoupling of programs that determine shoot and inflorescence meristem determinacy to specifically tune meristem transitions in different shoot systems.

Rare tomato cultivars with strongly branched inflorescences exist but are mainly grown for their aesthetic value. Cultivars such as Riesentraube (“giant bunch of grapes”) develop highly branched inflorescences with dozens of flowers but have been largely avoided by breeders due to low fruit set (Lippman et al., 2008). These natural *compound inflorescence* (*s*) mutants carry mutations in the homeobox transcription factor gene *S/SlWOX9*, a homolog of Arabidopsis *WUSCHEL-RELATED HOMEOBOX9*, *WOX9* (Lippman et al., 2008). Natural *s* mutants carry missense mutations in conserved residues of the S/SlWOX9 homeodomain that reduce S/SlWOX9 activity and cause the development of branched inflorescences and overproduction of flowers. At the molecular level, reduced *S/SlWOX9* activity is accompanied by the misexpression of hundreds of meristem stage-enriched genes (Park et al., 2012). This delay in meristem maturation causes individual apical meristems to release more than one lateral inflorescence meristem, which results in the development of branch points in the *s* mutant inflorescence (Lippman et al., 2008; Park et al., 2012). Complete loss of *S/SlWOX9* activity in apical meristems results in an arrest of meristem maturation and excessive overproliferation of inflorescence meristems on cauliflower-like inflorescence tissue, demonstrating that *S/SlWOX9* is essential for inflorescence meristem differentiation (Park et al., 2012; Rodríguez-Leal et al., 2017; Hendelman et al., 2021). *S/SlWOX9* activity is required for the proper expression of *ANANTHA (AN)*, which is a homolog of Arabidopsis *UNUSUAL FLORAL ORGANS* and encodes an F-box protein that interacts with the transcription factor FA to form a floral specification complex and trigger floral differentiation (Allen and Sussex, 1996; Lippman et al., 2008). Loss of *AN* activity refrains meristems from reaching floral identity and results in the formation of cauliflower-like inflorescence tissue.

The timely expression of *AN* depends on the activity of *TERMINATING FLOWER* (*TMF*), which encodes an ALOG (Arabidopsis LSH1 and Oryza G1) transcriptional regulator that directly represses *AN* expression (MacAlister et al., 2012; Huang et al., 2021a). Loss of *TMF* activity promotes a precocious expression of *AN* in transition meristems, which leads to a faster termination of primary shoot meristems and the development of single-flowered inflorescences (Figure 1; MacAlister et al., 2012). This accelerated meristem maturation program in *tmf* mutants involves precocious expression of additional floral meristem identity genes while transition meristem identity genes such as *S/SlWOX9* are not expressed, suggesting that *TMF* synchronizes meristem maturation and floral termination programs (MacAlister et al., 2012). Interestingly, the side shoots of *tmf* mutants develop regular multi-flowered inflorescences, indicating that *TMF* function is restricted to primary shoots and that redundant genes synchronize inflorescence meristem differentiation on axillary shoots. The tomato genome encodes 12 *ALOG*/*TMF FAMILY MEMBER* (*TFAM*) genes and the quadruple *tmf tfam123* mutant was shown to develop single-flowered inflorescences on all shoot systems (Huang et al., 2018, 2021b). Hence, at least four homologous *TFAM* genes are involved in the timely activation of *AN* for proper floral termination of axillary shoot meristems. The TMF protein was shown to physically interact with BLADE ON PETIOLE (BOP) transcriptional regulators (BOP1–3), which are co-expressed with *TMF* during vegetative meristem stages and decline toward floral identity (Figure 2; Xu et al., 2016). Furthermore, *bop123* triple mutants recapitulate the *tmf tfam123* quadruple mutant phenotype with single-flowered inflorescences on all shoots (Xu et al., 2016; Huang et al., 2021b). *BOP* genes have pleiotropic roles during inflorescence and leaf development and natural variation in *BOP* expression has been associated with differences in leaf complexity between wild and domesticated tomato species (Ichihashi et al., 2014; Wang et al., 2016). However, whether natural variation in *BOP* activity also affects inflorescence determinacy still remains to be determined.

### Suppression of tomato inflorescence branching during breeding

Although strongly branched inflorescences were largely avoided during breeding, there are reports of branched inflorescence mutants that arose by accident. During breeding for improved harvestability by removing the fruit abscission zones (joints), breeders reported that the causative *jointless2* (*j2*) mutation induces strong inflorescence branching and reduced fruit set in specific genetic backgrounds (Figure 4; Rick, 1956; Reynard, 1961). The *j2* mutation is caused by a *Rider* transposon insertion in a *SEPALLATA* (*SEP*) class MADS (*MINICHROMOSOME MAINTENANCE1* [*MCM1*], *AGAMOUS* [*AG*], *DEFICIENS* [*DEF*], *SERUM RESPONSE FACTOR* [*SRF*])-box transcription factor gene and induces inflorescence branching in genetic backgrounds that carry a secondary mutation in the homologous gene *ENHANCER OF JOINTLESS2* (*EJ2*) (Soyk et al., 2017a; Roldan et al., 2017). The secondary mutation is an intronic insertion in *EJ2* that arose early during domestication and causes partial mis-splicing of the *EJ2* transcript (Soyk et al., 2017a). The natural weak loss-of-function mutation (*ej2^W^*) results in flowers with elongated sepals while complete loss of *EJ2* activity from CRISPR null mutations lead to leaf-like sepals. Importantly, *j2* and* ej2* single mutants develop unbranched inflorescences, but epistasis between the homologous genes causes excessive inflorescence branching and low fertility in *j2 ej2* double mutants. Expression analyses of *j2 ej2* double mutants uncovered misexpression of meristem stage-enriched marker genes at transition and floral stages of meristem maturation suggesting that *j2 ej2* branching results from delays in meristem maturation (Soyk et al., 2017a). Mutations in the closely related MADS-box gene *LONG INFLORESCENCE* (*LIN*) also affect inflorescence architecture and lead to inflorescences with additional flowers and longer internodes that weakly branch. Triple *j2 ej2 lin* mutants give rise to inflorescence meristems that fail to achieve floral identity and produce cauliflower-like inflorescence tissue similar to the *an* mutant, demonstrating that *J2*, *EJ2*, and *LIN* genes synergistically regulate inflorescence complexity. Interestingly, the Arabidopsis genome encodes four *SEP* gene homologs, which redundantly regulate floral organ differentiation (Pelaz et al., 2000; Ditta et al., 2004). Hence, *SEP* MADS-box gene function diverged in tomato to regulate inflorescence architecture.

Breeders were able to combine *j2* and* ej2^W^* mutations by selecting additional genetic loci that suppress inflorescence branching (Figure 4) (Soyk et al., 2019). Modern jointless cultivars with both *j2* and* ej2^W^* mutations but unbranched inflorescences carry a tandem duplication that contains the *ej2^W^* splicing mutation. This increase in *ej2^W^* copy number results in higher levels of functional *EJ2* transcript and suppresses inflorescence branching. Remarkably, complete branching suppression is achieved by a second structural variant that affects a *TOMATO MADS-BOX3*(*TM3*)/*SUPPRESSOR OF OVEREXPRESSION OF CONSTANS1* (*SOC1*)-class MADS-box transcription factor gene (Alonge et al., 2020). Here, a reduction in copy number of *SISTER OF TM3* (*STM3*) leads to lower *STM3* expression and suppression of branching. Both *EJ2* and* STM3* copy number variants were present as cryptic variants in the domesticated tomato germplasm before *j2* and* ej2^W^* collided during modern breeding, illustrating how standing genetic variation contributes to breeding. Complete loss of *STM3* activity in CRISPR-induced *stm3* null mutants leads to late flowering indicating that *STM3* promotes meristem maturation toward the transition to flowering (Alonge et al., 2020). However, once the transition to flowering has been initiated, *STM3* activity represses meristem maturation of inflorescence meristems and allows inflorescence branching when the activity of *J2* and* EJ2* is absent. In Arabidopsis, mutations in the homologous gene *SOC1* also delay the transition to flowering but then promote the acquisition of floral identity (Liu et al., 2007, 2009). It is not fully understood how *STM3* functions both as promoter and repressor of meristem transitions, however, given that MADS-box proteins function in higher-order complexes, the interacting proteins are likely deciding.

### Exploiting inflorescence branching for improved productivity

Artificial selection for additional inflorescence branches led to yield increases during domestication and breeding of many crop species (Meyer and Purugganan, 2013). However, inflorescence improvement remains challenging in fruit crops such as tomato since strong inflorescence branching often causes low fruit set due to imbalanced source–sink relationships (Stephenson, 1981; Lippman et al., 2008). In tomato, this is illustrated by the natural double *j2 ej2* and single *s* mutants that develop strongly branched inflorescences with reduced fertility (Crane, 1915; Rick, 1956; Reynard, 1961). However, it has been recently shown that weakly branched inflorescences with high fertility can be obtained in tomato by fine-tuning the gene dosage of conserved meristem regulators. A quantitative reduction in MADS-box gene dosage in hybrids that are homozygous for *j2* mutations and heterozygous for weak *ej2^W^* mutations led to the development of weakly branched inflorescences (Soyk et al., 2017a). The forked inflorescences resulted in higher fruit yields since fruit set and size remained largely unaffected. Importantly, MADS-box genes have been shown to regulate inflorescence architecture also in Arabidopsis and rice, suggesting that homologous genes can be targeted for improved inflorescence architecture in both monocot and dicot crop species (Liu et al., 2013; Kobayashi et al., 2012). Increased fruit productivity from weak inflorescence branching was also achieved in hybrids that are heterozygous for natural mutations in *S/SlWOX9*, indicating that dosage-dependent regulators of meristem maturation are prime targets for tuning inflorescence architecture (Soyk et al., 2017a). Remarkably, the production of hybrids for reducing gene dosage from heterozygosity was bypassed by fine-tuning gene activity through modulating gene expression (Rodríguez-Leal et al., 2017). More specifically, CRISPR was used to randomly mutate the *cis*-regulatory regions upstream of *S/SlWOX9*, which allowed the production of novel weak loss-of-function *s/slwox9* alleles that cause a quantitative range in inflorescence branching. Similar approaches can likely be applied to conserved meristem regulators in other species. For example, the rice ALOG gene *TAWAWA1* regulates rice inflorescence (panicle) branching in a gene dosage-dependent manner (Yoshida et al., 2013). A better understanding of the genes and genetic networks that dictate meristem transitions will provide additional gene targets for precise tuning of inflorescence architecture to optimize flower, fruit, and seed production.

## Future perspectives: tuning meristem plasticity for climate-resilient agriculture?

The genetic networks that regulate meristem transitions are highly plastic and tightly linked to environmental changes (Andrés and Coupland, 2012). However, architectural plasticity was often mitigated during domestication and breeding to facilitate uniform plant growth and high productivity in new growth environments. In soybean, selection of natural mutations in the circadian clock gene *J* weakened the flowering response to inductive short-day conditions (Lu et al., 2017). The delayed transition to reproductive growth leads to larger plants with higher yields and allowed the expansion of soybean cultivation to tropical regions. In tomatoes, a *cis*-regulatory mutation in the antiflorigen gene *SP5G* reduces its inhibiting effects on flowering under long-day conditions and facilitated tomato cultivation in regions away from the equator (Soyk et al., 2017b; Zhang et al., 2018). However, architectural plasticity was not completely lost in crops and variation in plasticity exists within domesticated populations. In cucumber (*Cucumis sativus*), determinate shoot growth from a mutation in *CsTFL1* is modulated in a day-length sensitive manner by the homologous gene *CsTFL1d* (Wen et al., 2021). In tomato, multiple genetic loci were identified that are associated with adaptation of plant height, flowering time, and inflorescence architecture to fluctuations in temperature and water availability (Diouf et al., 2020). The underlying genes still require identification but may be harnessed for modulating architectural plasticity and adapting crops to new climatic conditions (see “Outstanding Questions”). Fine-tuning architectural plasticity could yield novel crop genotypes that are adapted to specific target environments and display optimized community performance (Weiner, 2019; Abbai et al., 2020).

Understanding the genetic changes that were selected by humans for adapting plants to new climatic regions can outline strategies for the development of novel genotypes for agriculture during climate change. Pan-genomes of crops and their wild ancestors allow the identification of genes and networks that were altered during domestication and breeding to modulate architectural plasticity. The standing genetic variation that is preserved in crop germplasms already presents a rich resource for adapting crops to new growth conditions. However, introducing genetic variation to new genotypic backgrounds can be challenging due to genetic linkage with deleterious alleles and often leads to unexpected phenotypic outcomes due to genetic interactions (Mackay, 2014). Genome editing allows the introduction of genetic variation in virtually any given background and overcomes negative effects from genetic linkage (Wallace et al., 2018). Precise targeting of conserved networks that underlie architectural plasticity could allow rapid crop adaptation to future growth environments and cultivation practices. For example, reducing day-length sensitivity and plant height could adapt fruit crops to cultivation in shorter growth seasons or restricted spaces for indoor agriculture (Gaston et al., 2020; Kwon et al., 2020). Genome editing also enables the direct introduction of agricultural traits into wild species (Gasparini et al., 2021). Such *de novo* domestication has been tested in wild relative species of tomato and rice (Li et al., 2018b; Zsögön et al., 2018; Yu et al., 2021). By targeting florigen pathway genes, wild tomato could be transformed into a more compact plant while pathogen resistance and salt tolerance were retained although background dependency has been observed (Li et al., 2018b). Genome editing can also be used to overcome genetic buffering from redundancy by targeting closely related genes and gene families. For example, simultaneous targeting of three tomato gibberellin receptors revealed gene redundancy during growth regulation that is lost under suboptimal environmental conditions (Illouz-Eliaz et al., 2019). Finally, genome editing of *cis*-regulatory regions allows the generation of quantitative phenotypic variation, for example, allelic series of domestication genes that can be used to fine-tune agricultural traits (Rodríguez-Leal et al., 2017). Together, these approaches could allow the generation of novel crop genotypes with custom plant architectures that are adapted to specific growth environments, presenting new avenues for breeding climate-ready crops.

**Outstanding questions**

- How are the effects of conserved meristem regulators modified in different genetic backgrounds, and what is the identity of the interacting genes?
- How do we uncouple the gene networks that regulate successive stages of meristem maturation to independently exploit shoot and inflorescence architecture for crop improvement?
- Is architectural diversity in crop populations mainly established by quantitative variation in a small number of conserved regulators or rather by variation in a large and diverse set of genes?
- What is the genetic architecture of plasticity in meristem development that relays environmental change to plant architecture?
- What is the impact of domestication and breeding on plasticity in meristem maturation?

## Notes

N.G., G.S., and S.S. conceived the content and wrote the article.

The author responsible for distribution of materials integral to the findings presented in this article in accordance with the policy described in the Instructions for Authors (https://academic.oup.com/plphys/pages/General-Instructions) is: Sebastian Soyk (sebastian.soyk@unil.ch).
